# Smartphone Apps to Support Self-Management of Hypertension: Review and Content Analysis

**DOI:** 10.2196/13645

**Published:** 2019-05-28

**Authors:** Tourkiah Alessa, Mark S Hawley, Emma S Hock, Luc de Witte

**Affiliations:** 1 Centre for Assistive Technology and Connected Healthcare School of Health and Related Research University of Sheffield Sheffield United Kingdom; 2 Biomedical Technology Department College of Applied Medical Sciences King Saud University Riyadh Saudi Arabia; 3 Health Economics and Decision Science School of Health and Related Research University of Sheffield Sheffield United Kingdom

**Keywords:** smartphone apps, mobile apps, self-management, hypertension, blood pressure, mobile applications

## Abstract

**Background:**

Hypertension is a widespread chronic disease, and its effective treatment requires self-management by patients. Health-related apps provide an effective way of supporting hypertension self-management. However, the increasing range and variety of hypertension apps available on the market, owing to the global growth in apps, creates the need for patients and health care professionals to be informed about the effectiveness of these apps and the levels of privacy and security that they provide.

**Objective:**

This study aimed to describe and assess all available apps supporting hypertension self-management in the most popular app stores and investigate their functionalities.

**Methods:**

In January 2018, the UK Apple and Google Play stores were scanned for all free and paid apps supporting hypertension self-management. Apps were included if they were in English, had functionality supporting hypertension self-management, and targeted adult users with hypertension. The included apps were downloaded and their functionalities were investigated. Behavior change techniques (BCTs) linked with the theoretical domain framework (TDF) underpinning potentially effective apps were independently coded by two reviewers. The data privacy and security of the apps were also independently assessed.

**Results:**

A total of 186 hypertension apps that met the inclusion criteria were included in this review. The majority of these apps had only one functionality (n=108), while the remainder offered different combinations of functionalities. A small number of apps had comprehensive functionalities (n=30) that are likely to be more effective in supporting hypertension self-management. Most apps lacked a clear theoretical basis, and 24 BCTs identified in these 30 apps were mapped to 10 TDF mechanisms of actions. On an average, 18.4 BCTs were mapped to 6 TDF mechanisms of actions that may support hypertension self-management behaviors. There was a concerning absence of evidence related to the effectiveness and usability of all 186 apps, and involvement of health care professionals in the app development process was minimal. Most apps did not meet the current standards of data security and privacy.

**Conclusions:**

Despite the widespread accessibility and availability of smartphone apps with a range of combinations of functionalities that can support the self-management of hypertension, only a small number of apps are likely to be effective. Many apps lack security measures as well as a clear theoretical basis and do not provide any evidence concerning their effectiveness and usability. This raises a serious issue, as health professionals and those with hypertension have insufficient information to make decisions on which apps are safe and effective.

## Introduction 

Internationally, hypertension is one of the most common chronic diseases in adults and is considered one of the main risk factors for numerous diseases such as stroke, heart disease, and renal failure [1]. It is estimated that around one billion individuals live with hypertension worldwide, and the majority of people are not proficient at controlling their blood pressure (BP) through medication or lifestyle choices, despite the fact that lowering BP decreases the risk of renal and cardiovascular disease [2]. Self-management is considered to be among the most effective methods of coping with hypertension, helping individuals with hypertension be more responsible for their own health [3].

The recent emergence of information and communication technologies such as mobile health supports the self-management of chronic conditions [4-7]. The increase in smartphone devices over the past decades has been rapid: By 2022, it is predicted that there will be 6.8 billion smartphone users [8]. This rapid increase of smartphone users corresponds with an increase in health apps offering health services and information [9,10].

Many apps have become available for patients with hypertension, and their number is increasing rapidly [11,12]. The majority of these smartphone apps are aimed at helping people manage and control their hypertension [11,12], but it is currently unclear to what extent the evidence supports their effectiveness. A recent systematic review of apps aimed at supporting the self-management of hypertension found few studies reporting the effectiveness of apps [13]. The majority of the apps in this review were study-specific and are therefore not available commercially in the app stores. The review concluded that apps containing more comprehensive functionalities, defined as three or more functionalities, are more likely to be effective in lowering BP [13] than apps with only one or two functionalities.

Even though many of the apps lacked evidence of theoretical underpinning, an examination of their functionalities revealed recognizable elements of behavioral change strategies [13]. Studies have shown that self-management programs are more likely to be effective if they are supported by theory-based interventions [14-16]. Theory allows identification of target behavior and strategies of behavioral changes needed to achieve desirable health outcomes. However, research has revealed that many commercial health apps lack theoretical underpinnings and theoretically consistent use of behavior change techniques (BCTs) [17-19]. In addition, the majority of health apps lack privacy and security measures that adequately ensure protection of users’ data, posing risks to user confidentiality [19,20]. This is problematic, as it compromises both the personal data of the user as well as their trust in the app.

These shortcomings might lead to significant concerns about apps having little to no benefit, or even presenting a risk to users [17], highlighting the necessity of providing adequate information about the effectiveness of these apps and the robustness of their privacy and security features for patients and health care professionals. As such, these findings increase the importance of characterizing and investigating potential theoretical mechanisms of action in existing commercial apps with comprehensive functionalities as well as assessing the privacy and security of such apps. A method of investigating potential theoretical mechanisms of action by grouping BCTs with theoretical domain framework (TDF) mechanisms of actions, using the TDF and BCT Taxonomy (v1) (BCTTv1), has been extensively employed to characterize BCTs in health interventions [17,21-23], especially those relating to chronic diseases.

A review by Kumar et al [12] searched for the most downloaded and popular apps in May 2014 and found there are many apps that support the self-management of hypertension by offering self-monitoring activities, feedback, reminders, and tailored information. However, the search was restricted to the 50 most popular apps for every search term (high blood pressure and hypertension) on the two smartphone platforms. As a result, only 200 apps were screened, excluding many apps that might be suitable to support people with hypertension in their self- management. Furthermore, this review excluded smartphone app–based BP-measuring devices, arguing that they lacked accuracy, despite evidence that some of these specific devices used for measuring BP have been found to be accurate [24,25].

This study has reviewed all the available apps, updated our knowledge of new apps related to hypertension, and described their main functionalities as well as functionality combinations. Even though apps have numerous potential benefits and are used by an increasing number of patients, to the best of our knowledge, no previous review has analyzed all available apps; considered functionality combinations; included apps associated with accessory devices; considered the link between BCTs and the TDF mechanisms of action, which underpin the potentially effective apps; and considered privacy and security assessment of the potentially effective apps. The aim of this study was to fill this knowledge gap by addressing these points.

## Methods

### Study Design

This study is a content analysis and review of apps supporting hypertension self-management available in the most popular app stores. The  *Quality and Risk of Bias Checklist for Studies That Review Smartphone Applications* [26] was utilized to ensure the adequate description of the app review’s methods.

### App Identification

#### Overview

In January 2018, an electronic search of apps was undertaken on the app stores of the two major types of smartphones in the United Kingdom—the iPhone (Apple Store) and Android (Google Play). These two platforms were searched because they were the world’s most used operating systems in 2017 [27]. The terms “hypertension” and “high blood pressure” were separately searched for in both stores. There were no restrictions concerning subcategories like “health and wellness.”

#### Inclusion and Exclusion Criteria

An app was included based on the following criteria: (1) The description was written in English, and “hypertension” or “high blood pressure” was included in the keywords or the accompanying description. (2) The collected data provided feedback, connected with health care professionals, or informed the patient about hypertension and self-management tasks related to hypertension; such tasks involve the self-monitoring of BP and other health data including healthy diet, exercising, taking medications, maintaining an appropriate weight, and managing stress. (3) The app was aimed at adults, in general, rather than health care providers (HCPs) specifically. Both paid and free apps were considered in the study.

An app was excluded based on one of the following criteria: (1) if it was not targeted at hypertension or if it focused solely on hypertension during pregnancy or primary prevention of hypertension; (2) if it was described in the app store catalogue as a “prank app” because it was not designed for medical purpose, but for entertainment; (3) if it was not designed for general use, for example, if it only provided services offered by particular hospitals or was designed only to be used as part of a specific study; and (4) if it did not run properly or required identification access after downloading the app, such as personal identification or primary care/hospital number.

The researcher selected the basic, completely functional version of an app if it had more than one version, such as high definition, lite, or pro. Apps appearing in both stores were rated independently to account for differences in features supported by various mobile operating systems. If an app appeared in response to searches for both “hypertension” and “high blood pressure” by a platform, it was included once, not twice.

### Screening and Selection of Apps

All apps that were identified through the search and met the inclusion criteria based on their title and description were downloaded by the researcher (TA) onto an iPhone 6 (running iPhone operating system, version 11.2.2; iOS, Apple Inc, Cupertino, CA) and Android Samsung Galaxy S7 (running 8.1 software; Seoul, South Korea). The apps were then screened for all exclusion and inclusion criteria. If they met the criteria, the apps were run for 2 days, so that the researcher could investigate all reminders or notifications that appeared. Data on all the included apps were charted.

### Data Abstraction

#### Overview

Abstracted data for all identified apps involved the name of the app, developer, version date, price and functions, available languages, and number of downloads. The involvement of health care professionals (eg, medical/health care professionals and behavior change specialists) was determined based on whether health professionals were involved in the development of the app as well as user involvement, which was included in the description on the app store. Following data abstraction, potentially effective apps (apps that were found to have comprehensive functionalities) were selected and considered for further analysis.

#### Functionalities

App functionalities were categorized based on the functionalities of hypertension self-management that have been determined in several previous studies about hypertension apps [11-13] and examined for effectiveness in scientific trials [28,29]. The functionalities that were considered in this study are self-monitoring, goal setting, reminders, educational information, feedback, stress management, communication with HCPs and others, and export of users’ data to others via email.

#### Apps Considered for Further Analysis

According to Alessa et al [13], apps with comprehensive functionalities are more likely to be effective. Such apps were identified on the basis of the presence of three or more functionalities, including (but not limited to) self-monitoring, reminders, and educational information or automatic feedback. Therefore, of the apps originally identified, apps that were found to have comprehensive functionalities were considered for further analysis. These apps were then analyzed to assess their privacy, security, and theoretical underpinning. This is because theoretical underpinning and privacy as well as security measures are essential criteria for apps to be used in health care [19].

##### Privacy and Security

Privacy and security were assessed based on the Online Trust Alliance [30] and the recommendations of the Information Commissioner’s Office [31]. These recommendations consist of seven questions examining the accessibility and availability of the privacy policy, the practices of data sharing and collecting, and data security as interpreted in the privacy and security statement (Table 3). These assessment questions and recommendations have also been previously used to assess privacy and security of existing health apps [19]. The assessment was conducted by two independent reviewers (TA and EH). Any discrepancies were resolved by discussion with the other researchers (LdW and MSH).

##### Theoretical Underpinning

To identify the mechanisms of action underpinning the existing apps with comprehensive functionalities, the BCT v1 Taxonomy (BCTTv1) was used to code the content of the app and extract the number of BCTs in each app and the frequency of use of each BCT in the apps. Each BCT was coded with “0” as Absent or “1” as Present [18]. The coding was undertaken by the two reviewers. Any disagreements were resolved by discussion with the other researchers. Interrater reliability for the presence or absence of the BCTs was assessed by calculating Cohen kappa for each item.

The present BCTs were then mapped to mechanisms of action of the TDF, based on several previously published expert consensuses linking BCTs to TDFs domains for health interventions, and the agreed judgement (consensus) of the study’s researchers [23,32-34]. The linking of BCTs to TDF was conducted independently by the two reviewers, and any discrepancies were resolved by discussion with other researchers of the study team. The final results were then agreed upon by the research team.

##### Additional Aspects

Two additional characteristics/aspects for the selected apps were also described—US Food and Drug Administration (FDA) or European Union Conformite Europeene (CE) approval and their individual user rating.

#### Statistical Analysis

The number and frequency of BCTs and TDF used in the reviewed apps were summarized as the SD, mean, and median using Microsoft Excel (Microsoft Corp, Redmond, WA). Proportions were also used to summarize the variables, including app functionalities, user ratings, and data privacy and security.

## Results

### Summary of Search Results

**Figure 1:**
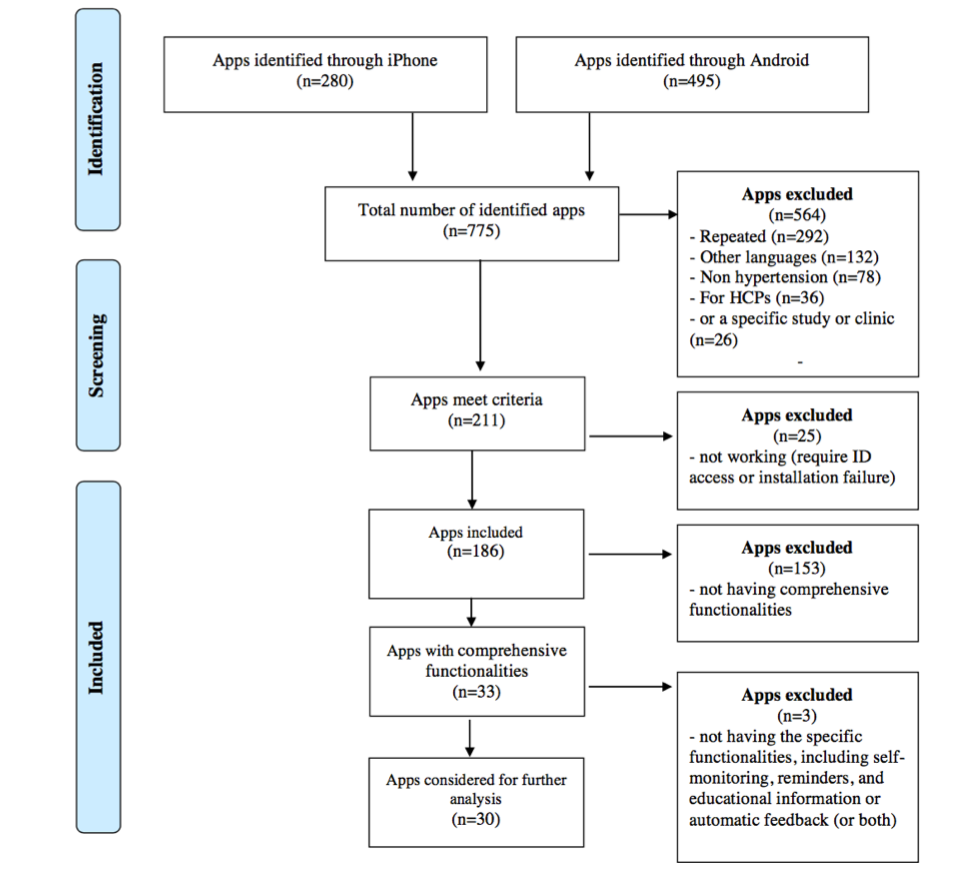
Flow diagram of the app-search process. ID: identification; HCP: health care provider.

The study steps are summarized in Figure 1. A search of the two app stores yielded a total of 775 apps (495 in Android Google Play Store and 280 in iPhone Apple Store). The titles and descriptions of these apps were screened for eligibility. A total of 564 apps were excluded because they did not meet the specific inclusion criteria. The 211 remaining apps (116 in Google Play and 95 in Apple Store) were considered for further analysis (installed). Subsequently, a total of 25 apps (11 in Google Play and 14 in App Store) were excluded because of registration problems (eg, requiring specific identification access such as hospital or primary care identification) or installation failure. The remaining 186 apps (106 in Google Play and 80 in App Store) were included in the review.

The cost of the apps varied. Over a quarter of the apps (27.9%) cost between £0.59 to £17. Most apps (134/186, 72.1%) were free to download. Of the apps that were free to download, 19 either were trials of the complete app or required subscription fees. All apps (n=186, 100%) were in English, although some also supported other languages such as Chinese, German, and Russian.

### General App Characteristics

Of the 186 apps that met the selection criteria, more than half (106/186, 57%) were available through the Android operating system. The remaining apps were available through the iPhone operating system (80/186, 43%). Only 11 apps were found to be available on both platforms (Multimedia Appendix 1).

The version date of the reviewed apps ranged from February 8, 2012, to February 13, 2018. According to the number of downloads, more than half of the included Android apps (60/106, 57%) had over 500 downloads. Information on the number of downloads was not available for Apple apps.

### Apps’ Purpose and Functionalities

All apps could be classified according to their functionalities, including stress management, communication with HCPs and others, self-monitoring abilities, reminders, automatic feedback, educational information, and goal setting. Each app had at least one of these functionalities (Multimedia Appendix 1).

Table 1 summarizes the frequency of functionalities across the included apps. The most common self-management functionality was educational information about hypertension (110/186, 59.1%). Educational content varied across apps. Most included basic educational information on high BP or information on diet and food (eg, dietary approaches to stop hypertension). Some apps contained general information on hypertension or alternative treatments. Although the majority of educational material was text-based, several apps contained video and images to depict their content.

The second most common functionality was self-monitoring (99/186, 53.2%), which allows users to monitor their BP and other data over a period of time presented in different forms, including graphs or tables, and to see an overview. The majority of these apps (n=94) aided BP tracking, while some of them also supported the self-monitoring of other data concerning medication, nutrition, physical activity, weight, and emotions. A few apps (n=5) only focused on tracking medication compliance, potassium intake, or sodium intake. Seven of these apps received BP readings automatically from the BP measurement device but do not provide a manual entry function. Of the remaining 84 apps, 73 necessitated manual entry of BP data, and 11 allowed both manual and automatic data transfer. Notably, a few apps (3/186, 1.6%) claimed that they turn the smartphone into a device capable of recording BP data. This was presumably achieved by using a “cuffless technique” in which the user puts a finger over the camera of their smartphone. Despite most of these apps claiming to measure BP, they did not report any evidence of their reliability and validity.

The third most common functionality was the provision of automatic feedback (52/186, 28%). This feedback was provided to users in different ways, either through self-care messages and notifications or by representing data in distinct color codes to inform the user of whether measurements have diverged from the average level.

One-fifth of the apps (39/186, 21%) had a functionality to remind users about BP measurements, their hospital appointments, their medication time(s), and personal goals. Certain apps (10/186, 5.4%) included BP goal setting, and a few also enabled the user to set other goals such as blood glucose levels, weight, and physical activity. A few apps (5/186, 2.7%) provided a tool for communication with others, including HCPs or friends, through text messaging, chats, or virtual meetings with coaches. Five apps (2.7%) also supported stress management by providing relaxation tips or other therapies.

Around one-fourth of the apps (51/186, 27.4%) allowed users to export their entered data over time directly to others, including physicians, via email and other apps such as “WhatsApp,” thus facilitating patient-physician communication.

As shown in Table 2, the majority of the apps (n=108) included only one functionality such as educational information (n=82), self-monitoring (n=25), or stress management (n=1). Almost one-fourth (45/186, 24.1%) of the apps combined two functionalities, while a small number of apps (33/186, 17.8%) included comprehensive functionalities (ie, three or more functionalities). None of the 33 apps included all 8 abstracted functionalities. Thirty of these apps included, among other functionalities, self-monitoring and reminders, with educational information (5/186, 2.7%), automatic feedback (16/186, 8.6%), or both (9/186, 4.8%).

**Table 1:** Frequency of app functionalities.

Functionality	iPhone (Apple; N=80), n (%)	Android (Google Play; N=106), n (%)	Total (N=186), n (%)
Educational information	34 (43.8)	76 (70.8)	110 (59.1)
Self-monitoring	56 (70)	43 (40.6)	99 (53.2)
Feedback	36 (43.8)	16 (15.1)	52 (28)
Export	29 (36.3)	22 (20.8)	51 (27.4)
Reminder	23 (28.8)	16 (15)	39 (21)
Goal setting	8 (10)	2 (1.9)	10 (5.4)
Stress management	3 (3.8)	2 (1.9)	5 (2.7)
Communication with others	4 (5)	1 (0.9)	5 (2.7)

**Table 2:** Common combinations of app functionalities.

Functionality combinations	Number of functionalities	iPhone (N=80), n (%)	Android (N=106), n (%)	Total number of apps used in combination, n (%)
Educational informational	1	21 (26.3)	61 (57.5)	82 (44.1)
Self-monitoring	1	10 (12.5)	15 (14.1)	25 (14)
Stress management	1	1 (1.3)	0 (0)	1 (0.5)
Self-monitoring + Feedback	2	18 (22.5)	6 (5.7)	24 (12.9)
Self-monitoring + Educational information	2	3 (3.8)	6 (5.7)	9 (4.8)
Self-monitoring + Reminder	2	5 (6.25)	3 (2.8)	8 (3.8)
Educational information + Communication with others	2	2 (2.5)	0 (0)	2 (1.1)
Educational information + Stress management	2	0 (0)	1 (0.9)	1 (0.5)
Educational information + Reminders	2	0 (0)	1 (0.9)	1 (0.5)
Self-monitoring + Reminder + Feedback	3	5 (6.3)	3 (2.8)	8 (4.3)
Self-monitoring + Reminder + Educational information	3	2 (2.5)	3 (2.8)	5 (2.7)
Self-monitoring + Feedback + Communication with others	3	1 (1.3)	1 (0.9)	2 (1.1)
Self-monitoring + Reminder + Feedback + Goal setting	4	5 (6.3)	2 (1.9)	7 (3.8)
Self-monitoring + Reminder + Feedback + Educational information	4	3 (3.8)	3 (2.8)	6 (3.2)
Self-monitoring + Feedback + Educational information + Goal setting	4	1 (1.3)	0 (0)	1 (0.5)
Self-monitoring + Reminder + Feedback + Goal setting + Communication with others	5	1 (1.3)	0 (0)	1 (0.5)
Self-monitoring + Reminder + Feedback + Educational information + Stress management	5	1 (1.3)	1 (0.9)	2 (1.1)
Self-monitoring + Reminder + Feedback + Educational information + Stress management + Goal setting	6	1 (1.3)	0 (0)	1 (0.5)

The most frequently used combination of functionalities was self-monitoring with automatic feedback (24/186, 12.9%). The second most common combination was self-monitoring and educational information (9/186, 4.8%).

### Involvement of Health Care Professionals and Users in App Development and Scientific Evaluation

Six apps (3.2%) claimed to have had contributions from an HCP or medical organizations during their development; the other apps did not. No apps reported end-user involvement (eg, hypertensive patients) in their development. None of the apps appeared to have been scientifically evaluated. The description provided indicates that there is an absence of evidence concerning the effectiveness or usability of apps designed to help manage hypertension.

### Data Security and Privacy

#### Accessibility and Availability of Privacy Policy

Of the 30 apps in the study that had comprehensive functionalities, the availability of a privacy policy in English was found in 20 apps (66.6%; Table 3). Of the 10 apps without an English-language privacy policy, only one provided a link to such a policy, but the link was not functional. Further, 4 of these apps provided a privacy policy in non-English languages.

The short-form notice indicating key data practices was not applicable to the 20 apps that provided a privacy policy, since the policies were already concise. Apps rarely offered multilingual policies, with only one app offering a policy in two other languages.

**Table 3:** Data privacy and security assessment of apps (data gathering, sharing, and security) on the basis of the description in the privacy policy.

Privacy and security question		iPhone (N=12), n (%)	Android (N=8), n (%)	Total (N=20)^a^, n (%)
**Is the privacy policy available without the need to download the app?**				
	No	0 (0)	0 (0)	0 (0)
	Yes	12 (100)	8 (100)	20 (100)
**Is the privacy policy available within the app?**				
	No	5 (42)	2 (25)	7 (35)
	Yes	7 (58)	6 (75)	13 (65)
**Is there a short form notice (in plain English) highlighting key data practices?**				
	No	0 (0)	0 (0)	0 (0)
	Yes	0 (0)	0 (0)	0 (0)
	Not applicable	12 (100)	8 (100)	20 (100)
**Is the privacy policy available in any other languages?**				
	No	11 (92)	8 (100)	19 (95)
	Yes	1 (8)	0 (0)	1 (5)
**Does the app collect personally identifiable information?**				
	No	1 (8)	0 (0)	1 (5)
	Yes	10 (83)	6 (75)	16 (80)
	Not specified	1 (8)	2 (25)	3 (15)
**Does the app share users’ data with a 3rd party?**				
	No	0 (18)	0 (11)	0 (15)
	Yes	8 (67)	6 (75)	14 (70)
	Not specified	4 (33)	2 (25)	6 (30)
**Does the app say how the users’ data security is ensured? For example, encryption, authentication, and firewall**				
	No	6 (50)	5 (62)	11 (55)
	Yes	6 (50)	3 (38)	9 (45)

^a^Only 20 apps had a privacy policy; 10 apps did not have a privacy policy available*.*

#### Data Gathering and Sharing

Sixteen of the 20 apps with a privacy policy in English (80%) disclosed the collection of personally identifiable information such as age. In three other apps, the data-gathering practices were not discussed. One app did not report personal data gathering.

The developers of 14 apps revealed that they shared the data they gathered with third parties and discussed sharing practices. In three apps, data-sharing practices were not discussed. In three other apps, the policies stated that data would not be shared, except in exceptional cases that were general and vague. Despite reporting that they did not share data, except in exceptional circumstances, we believe that they share data without specifically discussing their data-sharing practices.

#### Data Security

Almost half (9/20) of the apps reported how consumer data were secured. In these cases, the privacy policies explained that data safety and security are essential to their practices and that users’ data have been encrypted, anonymized, or accessed only by authorized persons.

### Behavior Change Techniques and Theoretical Domain Framework

#### Presence of Behavior Change Techniques

We identified 24 BCTs in the 30 reviewed apps featuring comprehensive functionalities (Table 4). The Cohen kappa for agreement between the two reviewers for coding BCTs was 0.85.

The total number of BCTs in each app ranged between 6 and 17 BCTs, with a mean of 18.4 (SD 2.6) and a median of 9. The most frequently used BCTs were “Self-monitoring of behavior,” “Prompts/cues,” and “Action planning.” These were present in all 30 reviewed apps. The next most frequently used BCTs were “Feedback on behavior” and “Monitoring of behavior by others without feedback,” which were present in 25 and 24 apps, respectively. Two of these 24 BCTs (“Social comparison” and “Demonstration of the behavior”) were present only once. Table 4 presents the frequency of BCTs used in these 30 apps.

**Table 4:** Behavior change techniques (N=24) used in the reviewed apps (N=30).

Behavior change technique	Most common function of the app	Number of apps
Self-monitoring of behavior	Allows users to frequently record and self-monitor the performed behavior of their health in a diary, including measuring BP^a^, weight, emotion, and record if they took medication or other behaviors	30
Prompts/cues	An alarm is activated when it is time to perform a task with the purpose of cueing and promoting the behavior	30
Action planning	Setting a reminder to perform a task(s) (taking medication, measuring BP, exercise, etc) at a specific time	30
Feedback on behavior	Provides feedback on the entered data by representing data in different color codes or through self-care messages	25
Monitoring of behavior by others without feedback	Allows others to consensually observe the performed behavior to support the management of hypertension	24
Self-monitoring of outcomes of behavior	Allows users to monitor BP readings (eg, average) and view trends over time	22
Feedback on outcomes of behavior	Provides feedback on behavior outcomes over time (eg, average) to allow users to view their health status	17
Pharmacological support	Setting a specific reminder to facilitate medication adherence at a specific time	15
Information about health consequences	Offers educational information about the health benefits and consequences of controlling and managing hypertension	14
Instruction on how to perform a behavior	Provides overall orientation on hypertension management (including how to self-monitor BP) as well as other behaviors	10
Focus on past success	Offers the number of cases where the BP level has been successfully controlled or the successful achievement specific goals	10
Credible source	Contains information from credible sources	9
Goal setting (outcome)	Allows users to set health goals for controlling BP	8
Problem solving	Enables users to analyze BP with other behaviors trends and develop the knowledge required to understand how to achieve optimal BP level by identifying problems that hinder BP control	8
Goal setting (behavior)	Allows users to set goals for the behavior to be attained, including times to measure BP, weight, exercise, and food goals	7
Review outcome goal(s)	Allows users to examine how well the BP level was controlled according to the agreed goal and consider modifying outcome goals accordingly	5
Review behavior goal(s)	Allows users to modify their goals according to their achievements	5
Social support (unspecified)	Offer a space to chat with others (eg, friends or families)	3
Social incentives	Add points or provides badges for users when achieving their target goals and tasks	3
Salience of consequences	Shows pictures of health consequences such as dizziness to shed light the dangers of uncontrolled BP	2
Monitoring of emotional consequences	Allows users to record their feeling after performing tasks including measuring BP or exercises	2
Reduce negative emotions	Provides advice on the ways to minimize negative emotions	2
Demonstration of the behavior	Offers an observable sample of the performance of the behavior with the help of pictures for the person to aspire to or imitate	1
Social comparison	Allows comparison of the user’s own performance with others by sharing his/her performance to draw attention to the performance of others	1

^a^BP: blood pressure.

#### Mechanisms of Action of the Theoretical Domain Framework

BCTs present in the 30 reviewed apps could be linked to 10 TDF mechanisms of action. The number of TDF mechanisms of action underlying each app varied, ranging from 5 to 9, with a mean (SD) of 6 (1) and a median of 6.

The most common TDF mechanisms of action were “Behavior regulation” (30/30, 100%), “Knowledge” (30/30, 100%), “Goals’ (30/30, 100%), “Memory attention and decision process” 30/30, 100%), and “Beliefs about consequences” (30/30, 100%), which were present in all studied apps. The “Behavior regulation” mechanism of action was typically targeted by the “Self-monitoring of behavior” and “Self-monitoring of outcome(s) of behavior”BCTs, while “Knowledge” was mostly targeted by “Feedback on outcome(s) of behavior,” “Instruction on how to perform the behavior,” “Information about health consequences,” and “Feedback on behavior.” The “Goals” and “Memory attention and decision process” were mostly targeted using “Action planning” and “Prompts/cues” BCTs, respectively. The next most common mechanisms of action identified were “Beliefs about capabilities” (16/30), which was mostly targeted using BCTs “Problem solving,” “Focus on past success,” and “Social incentive.” Almost one-third of the apps (9/30) had “Skills” as a mechanism of action, which was mostly targeted using “Problem solving” and “Demonstration of the behavior.” “Social influences” (4/30 13%) was an infrequently used mechanism of action. The least common mechanisms were “Reinforcement” (3/30, 10%) and “Emotion” (3/30, 10%), which were present in only three apps. The mechanisms of action “Intention,” “Optimism,” “Professional role and identity,” and “Environmental context and resources” were not presented in any app.

#### Additional Aspects

None of the 30 apps were FDA or CA approved. Eighteen apps (60%) were found to have information available concerning their user rating. Of these 18 apps, 13 (72.2%) scored 4 or more stars (of 5). Only 5 (27.8%) app ratings were below 4 (Multimedia Appendix 1).

## Discussion

### Principal Findings

This study aimed to review all apps developed to support the self-management of hypertension, which are available on the two most popular app stores Google Play (Android) and Apple Store (iPhone).

The review showed that a significant number of apps (n=186) are available to support the self-management of hypertension. These apps had similar functionalities, although they differed in terms of the combination of functionalities provided. The majority of these apps had only one function (n=108, 58.1%), while the remaining offered different combinations of functionalities. This review indicated that there were few apps with comprehensive functionalities. Apps with comprehensive functionalities are potentially more effective [13].

There are also serious issues regarding the privacy and security of the apps and inconsistencies in apps’ theoretical underpinning, where in many cases, apps were developed without an explicitly clear theoretical basis. The evaluation of the selected apps’ data security and privacy revealed that the privacy policy was not available for 35% of the apps assessed in detail. Most apps gathered identifiable personal information and engaged in sharing user data with third parties and almost half of the selected apps (45%) did so without clearly disclosing how data security was ensured. The evaluation of the theoretical underpinning of apps revealed that a total of 24 BCTs, ranging from 6 to 17 (with median of 9), identified in the 30 reviewed apps mapped to 10 TDF mechanism of actions, ranging from 5 to 9 (with a median of 6), may have supported hypertension self-management behaviors. These findings are similar to reviews of apps related to other chronic diseases that have reported that few apps contain both comprehensive functionalities [10,35] and inconsistent BCTs [17,21]. Despite much research that code BCTs underpinning health apps [17,19,21,22], there is little research reviewing how existing apps map BCTs to TDF domains. The linking of BCTs to TDF conducted in the present study may help developers and researchers in selecting appropriate BCTs when developing apps. It may also help researchers understand which BCTs are effective and how they exert their effects [36-38].

None of the reviewed apps made claims based on behavioral theories or strategies relating to various self-management interventions, although self-management programs are likely to be effective if they are supported by theory-based interventions [14-16]. This may be because the expertise of health professionals was not factored into the development of the majority of these apps [35,39], despite the stressed importance of involving multidisciplinary perspectives and skills in developing a product within a user-center design framework [19]. However, for the 30 reviewed apps with comprehensive functionalities, the examination of the BCT and TDF domains underpinning them shows that a number of BCTs and TDF mechanisms of action were present. There is still no conclusive evidence for which combinations of BCTs or TDFs are the key moderators for effective chronic disease self-management, especially hypertension [21,40,41]. This is an area that requires further research. However, all present TDFs in these 30 apps have the potential to stimulate hypertension self-management activities through different mechanisms of action, particularly those of “Behavior regulation,” “Knowledge,” “Memory, attention and decision processes,” and “Goals.” These mechanisms of action are supported by studies identifying the key TDF domains that need to be targeted to influence patient behaviors and support self-management in chronic diseases [42,43]. Although other studies have also found that “Skills,” “Emotions,” “Reinforcement,” and “Belief of capabilities” are essential to increase people’s motivation in managing their health, many of the reviewed apps lack these characteristics [44].

The evaluation of the privacy policy showed that the security and privacy of consumers could be substantially improved. Our findings are in accord with those of earlier studies that have evaluated data security and safety of existing apps [19,20]. Huckvale et al revealed that one-fifth of apps in the National Health Service Apps Library lacked privacy policies, and the majority of the apps violated user data privacy and security [20]. Practices of data gathering and analysis by app developers can be advantageous to users, providing greater levels of personalization and data-informed improvements to the app. However, such practices of data gathering should be disclosed clearly, so that a potential user is aware of the possible risks to their data security [45]. To ensure users are able to make fully informed decisions, they must be equipped with the skills and information necessary to scrutinize these privacy and security policies. Because of the large scale of the app market, the regulation and preservation of data protection is difficult. As a result, the management of data privacy and security is entrusted to the developers of apps [46].

This review identified a small number of apps that are able to use smartphones as a medical device (cuff-less device) to measure BP. However, none of these apps were approved as a validated medical device. Indeed, cuff-less devices for measuring BP based on smartphone apps have recently been shown to be highly inaccurate and unfeasible [47] and may negatively affect patients’ health and safety. This is of particular concern, since a recent study by Kumar et al (2016) found that even though only a small number of apps have this feature, users have a strong inclination to download and favorably rate these types of apps [12]. This highlights the need for extensive clinical validation studies in different patient populations before such technology is used in commercial and clinical capacities. As such, physicians should currently be aware of the use of such apps by their patients and should promote only the use of validated devices for BP measurement.

Apps with more comprehensive functionalities have the potential to be more difficult for patients to use. This study found that there was an absence of evidence concerning the usability of the apps in the apps’ descriptions. Although this study did not directly evaluate the apps’ usability, user ratings were used as a proxy of the apps’ usability. The usefulness of user ratings as a measure of apps’ technical usability is questionable. In a review of mobile apps for the self-management of diabetes, Hood et al [48] found that the user rating was poorly correlated with the results of the study’s usability evaluation. However, in a general sample of health apps, Mendiola et al found that user ratings could be related to an app’s technical usability regarding aspects such as layout, interactive features, and general ease of use [49]. The user ratings for the apps considered in this study were high, with 73% obtaining 4 or more stars. This is in line with previous studies [50,51] where participants reported that they were satisfied with apps that include comprehensive functionalities, finding them easy to use.

The majority of apps identified in the recent systematic review of Alessa et al [13] were study specific, that is, developed for the aims of the study alone [13]. However, the apps considered in this review were commercially available apps in app stores. The descriptions of these apps lacked evidence about their effectiveness and did not even mention or consider the importance of such evidence. None of them were approved by the FDA or CE as a medical device. This is in line with previous reviews, which reported that the rapid growth of the commercial market for such apps has created an overabundance of apps that lack readily available evidence of their effectiveness [25] and lack FDA or CE approval [52,53]. Applying the findings of this recent systematic review within this review of commercial apps indicated that few apps (30/186) seem to have the potential to be effective. Apps that have this potential would need to be scientifically evaluated to ensure that this potential for effectiveness and usability is realized in practice. This indicates a critical gap between the research domain and the work of commercial app developers, emphasizing the importance of cooperation between them.

### Limitations and Strengths of the Study

This review has a number of limitations. First, the review only included apps that were developed to be used by English-speaking users, excluding apps in other languages such as Chinese. Second, since these apps are tailored for the self-management of hypertension, they support a wide range of different behaviors such as medication adherence, weight, diet, and physical activity in addition to the self-monitoring of BP, which makes it challenging to code them according to a single specific behavior and exclude other behaviors. This may be attributed to the complexity of the self-management process, which encompasses an array of behaviors and activities to effectively control BP. Third, this study excluded apps that require identification access. Moreover, the content of educational information of included apps was not checked to ensure they were up to date and met medical standards and health literacy guidelines. Finally, data privacy and security were assessed in relation to policy statements rather than practices. There is evidence of inconsistencies in some cases between the real practices of app developers and policy statements [20].

Despite these limitations, the study has several strengths. As this study reviewed all apps supporting the self-management of hypertension, rather than limiting itself to only the most popular apps [12], the results of this review offer a general picture of the present status of smartphone app stores in the field of hypertension. This comprehensive review will guide further research and development of these tools in different ways, for example, by encouraging developers and researchers to assess commercially available apps’ effectiveness and usability among potential users and by urging app developers to be more transparent about privacy and security. The study reviewed apps on the two most common smartphone platforms; it thus considered a large user base. Furthermore, this study is the first systematic review to explore the theoretical underpinning of apps by seeking to map BCTs to TDF domains in apps containing comprehensive functionalities. The insights could be useful for content developers designing apps in the area of hypertension or other chronic diseases that aim to engage both users and health care personnel who are likely to encourage patients to utilize these technologies.

### Recommendations

Based on the result of this review, some recommendations can be made. Despite the widespread availability of apps, potential users and health care providers should be made aware of the shortcomings in the security of private data as well as in the potential effectiveness of the apps in supporting hypertension self-management. Future efforts (and collaborations) should also be made by both researchers and commercial developers to encourage the development of apps that demonstrate scientific evidence of their effectiveness and usability to the public. The importance of involving end users in app development should be noted, as it helps improve user satisfaction and acceptance. This study’s findings encourage further research to evaluate app effectiveness and technical usability. It is important to assess the effectiveness of commercially available apps in order to determine the positive and potential negative effects of using the app.

### Conclusions

The review identified the widespread accessibility and availability of smartphone apps with a range of combinations of functionalities that can support the self-management of hypertension. However, relatively few of these apps contained comprehensive functionalities, which are more likely to be effective in lowering blood pressure; many lacked security measures; and most lacked a clear theoretical basis. Furthermore, there is a concerning absence of evidence with regard to their effectiveness and usability and involvement of health care professionals in the development process. This raises a serious practical issue for health care professionals and patients in determining which app to choose or use, as there are no specific criteria for them to make an informed selection. These findings demonstrate that the technical usability and effectiveness of apps in supporting the self-management of hypertension urgently need to be evaluated and that clear criteria need to be established to guide the selection of the most suitable app.

## Appendix

Multimedia Appendix 1 Data of the included apps.

## Abbreviations

BCT: behavior change technique
BCTTv1: behavior change technique taxonomy v1
BP: blood pressure
CE: European Union Conformite Europeene
FDA: Food and Drug Administration
HCP: health care provider
TDF: theoretical domain framework
