# Supplementary material for: Smartphone Apps to Support Self-Management of Hypertension: Review and Content Analysis
Source: JMIR Mhealth Uhealth. 2019 May 28;7(5):e13645. doi: 10.2196/13645 (PMC6658295; doi:10.2196/13645)
Supplement: Multimedia Appendix 1 [file mhealth_v7i5e13645_app1.pdf]

App name	App store	The developer	Version date	Cost	Number of downloads	User ratin gs	Privacy & Security	App Functionalities							
								Self-monitoring	Reminde r	Educational information	Feedbac k	communication with others	Stress managemen t	Goal setting	Export
1. Accutension Stetho	Apple	Shangahi Hulu Device Co Ltd	19/11/2017	Free	NA[c]	–[d]	–	✔							
2. AGR blood pressure Log	Apple	Angel Garcia rubio	30/11/2016	1.99	NA	–	–	✔			✔				✔
3. Best Way to Lower Your High Blood Pressure Fast and Early Prevention Guide & Tips for Beginners	Apple	Alex Baik	-	2.99	NA	–	–			✔					
4. BGL smart sphygmomanometer	Apple	FIRST RANK tech	5/1/2017	Free	NA	–	–	✔			✔				
5. Blood Pressure – SmartBP	Apple	Evolve medical System, LLC	8/11/2017	Free (5.99)[a]	NA	4.3	Y[e]	✔	✔		✔			✔	✔
6. Blood pressure & plus diary	Apple	Jan-Hendrik Damerau	4/11/2017	0.99	NA	NR[b]	N[f]	✔	✔		✔				✔
7. Blood Pressure Companion	Apple	Maxwell Software	20/12/2017	Free (3.99)[a]	NA	4.7	Y	✔	✔		✔				✔
8. Blood pressure diary	Apple	Openit Inc.	12/2/2018	Free	NA	NR	Y	✔	✔		✔			✔	
9. Blood pressure Diary 2	Apple	cellHigh	8/10/2016	4.49	NA	–	–	✔			✔				✔
10. Blood pressure Grapher	Apple	Ai Imai	11/12 2017	Free (9.99)[a]	NA	–	–	✔							
11. Blood pressure Lite-BP Tracker for Hypertension	Apple	Codulis	3/3/2016	Free (4.99)[a]	NA	–	–	✔			✔				✔
12. Blood Pressure monitor – cloud Ed	Apple	Thomas Kress	24/9/2015	1.99	NA	–	–	✔			✔				✔
13. Blood Pressure Monitor - Health Edition	Apple	Thomas Kress	23/5/2015	2.29	NA	–	–	✔			✔				✔
14. Blood pressure PRO with on	Apple			9.99	NA	–	–	✔			✔				✔
15. Blood pressureDB	Apple	klier.net	26/5/2016	Free	NA	–	–	✔	✔						
16. Bloodnote – blood pressure control	Apple	Matt Ludzen	23/1/2015	1.99	NA	–	–	✔			✔				

17. BP Assistant – Blood pressure	Apple	Josef Moser	2/8/2017	2.99	NA	–	–	✔							✔
18. BP Grapher: Simple	Apple	Ai Lmai	4/1/2018	Free (2.99)[a]	NA	NR	Y	✔	✔		✔			✔	✔
19. BP Log	Apple	ser.soft GmbH	22/11/2017	17.0	NA	–	–	✔							
20. BP Matters	Apple	Nanyang Polytechnic	10/11/2016	Free	NA	NR	N	✔	✔		✔				
21. BP Tracker – Smart Blood Pressure	Apple	Guangodong Bioloight Meditech co., Ltd.	19/9/2017	Free	NA	–	–	✔			✔	✔			
22. bp Trax® – Blood Pressure Treatment Tracker, Lifestyle, Resting, BMI, MAP, Pulse Rate	Apple	IMobLife Inc	25/6/2015	1.99	NA	–	–	✔			✔				
23. BP Wiz Pro – Blood pressure & Drug	Apple	LinkLinks LTD	30/6/2017	1.99	NA	3.9	Y	✔	✔		✔				✔
24. BPMon Pro-Blood Pressure Monitor	Apple	Anatoly Butko	18/4/2017	3.99	NA	–	–	✔			✔				✔
25. BP-Monitor Blood Pressur	Apple	Adappt LLC	15/10/2017	Free	NA	–	–	✔			✔				✔
26. Braun Healthy heart	Apple	Kaz US, Inc	31/10/2015	Free	NA	3.0	Y	✔	✔	✔	✔		✔		
27. Caracal	Apple	Caracal	12/10/2017	Free (12.99)[a]	NA	–	–	✔							
28. Control tension	Apple	Les Laboratories service	15/5/2015	Free	NA	NR	N	✔	✔	✔					✔
29. Cora – Blood Pressure	Apple	Swiftware	21/11/2017	Free (2.79)[a]	NA	4.4	Y	✔	✔	✔	✔		✔	✔	✔
30. Dash diet food	Apple	Mark Patrick Media	12/11/ 2016	2.29	NA	–	–			✔					
31. Dash Diet guide	Apple	Prestige Worldwide Apps LLC	30/9/2015	Free	NA	–	–			✔					
32. Dash Diet Plan - Lower High Blood Pressure Naturally With Dash Diet!	Apple	nipon phuhoi	-	9.99	NA	–	–			✔					
33. Dash diet recipes and more	Apple	Becky Tommervik	-	1.09	NA	–	–			✔					
34. DASH-Sodium Diet App Bundle	Apple	First Line Medical Communications Ltd	-	7.99	NA	–	–	✔		✔					

35. Diet and Hypertension	Apple	SoftPsych LLC	-	4.99	NA	–	–			✔					
36. Easy blood pressure	Apple	Seaside apps	14/1/2016	0.99	NA	–	–	✔							✔
37. Easy Blood Pressure Diary	Apple	SILECI	23/1/2016	Free	NA	–	–	✔							
38. ESH Care	Apple	YouCo	2/12/2015	Free	NA	NR	Y	✔	✔	✔					
39. Fast BP –Blood Pressure Log & Track	Apple	Christion Richert	18/12/2015	0.99	NA	3.7	N	✔	✔		✔			✔	✔
40. HeartStar BP Monitor	Apple	Pattern Health	19/10/2017	Free	NA	3.8	Y	✔	✔		✔			✔	✔
41. Hemie	Apple	Creative Studio	-	Free	NA	NR	N	✔	✔	✔	✔				
42. High Blood Pressure Symptoms	Apple	Chris Bechard		Free (1.99)[a]	NA	–	–			✔					
43. High blood pressure Symptoms	Apple	Chris Bechard	-	Free (2.29)[a]	NA	–	–			✔					
44. High blood pressure Symptoms & BP	Apple	Venture Technology Ltd	28/11/2015	Free	NA	–	–			✔					
45. High blood presure	Apple	Personal Remedies, LLC	17/6/2016	3.49	NA	–	–			✔					
46. HoMedic	Apple	HoMedics	3/11/2016	Free	NA	1.5	Y	✔	✔	✔	✔				
47. How to lower blood pressure	Apple	globalappz	22/9/2016	3.49	NA	–	–			✔					
48. Hypertension BP	Apple	Manal Abuelazem	-	2.99	NA	–	–			✔					
49. HypertensionHealth	Apple	Les Labortires Service	-	Free	NA	–	–			✔		✔			
50. iBP Blood Pressure	Apple	Leading Edge Apps LLC	16/5/2016	0.99	NA	–	–	✔			✔				✔
51. iFORA BP	Apple	ForaCare Inc.	18/11/2017	Free	NA	–	–	✔			✔				✔
52. Iscian health	Apple	HONSUN (Nantong) Co., ltd	17/11/2016	Free	NA	–	–	✔			✔				
53. John Carberry high blood pressure	Apple	John Carberry	18/2/2016	4.49	NA	–	–			✔					
54. Kang BP	Apple	kangkang	9/12/2015	Free	NA	NR	N	✔	✔		✔	✔		✔	
55. LifeCourse HyTen	Apple	PInCH Medical Systems Limited	26/10/2016	Free	NA	NR	Y	✔	✔	✔	✔				

56. Low Sodium Foods	Apple	Nasir Hussain	5/6/2017	Free	NA	–	–			✔					
57. Managing blood pressure	Apple	Semos & Co.	1/7/2016	4.49	NA	–	–			✔					
58. MedM Blood Pressure	Apple	MedM Inc	21/11/2017	Free	NA	–	–	✔			✔				✔
59. Medmobile (en)	Apple	medmobile	27/10/2017	Free	NA	–	–	✔			✔				
60. Microlife Connected Health	Apple	Microlife Corp.	19/8/2017	Free	NA	–	–	✔							✔
61. Monitor My BP	Apple	APG Solutions, LLC	3/11/2017	Free (0.99)[a]	NA	–	–	✔	✔						✔
62. My Blood Pressure Readings	Apple	Puig Labs	31/3/2016	Free	NA	–	–	✔	✔						✔
63. My medication journal	Apple	Ara Wolf	11/4/2015	0.99	NA	–	–	✔	✔						
64. MyBloodWorks	Apple	Gogle LLC.	18/9/2015	3.49	NA	–	–	✔							
65. Naturally Reduce Blood Pressure Guide and Tips	Apple	Xin Tan		3.49	NA	–	–			✔					
66. Normal Blood pressure - How to reduce	Apple	Venture Technology	25/5/2017	Free	NA	–	–			✔					
67. Nutrition Hypertension	Apple	BulitByDoctors	8/1/2017	Free	NA	–	–			✔					
68. Nutrition Wise	Apple	Consultation Massawippi	22/9/2017	2.29	NA	–	–			✔					
69. Potassium Counter & Tracker for Healthy Food Diets	Apple	First Line Medical Communications Ltd	5/7/2017	4.99	NA	–	–	✔		✔					
70. Pukono	Apple	Fundació Althaia	13/7/2017	Free	NA	–	–			✔					
71. Qardio heart health	Apple	Qardio, Inc.	17/6/2016	Free	NA	NR	Y	✔	✔		✔				✔
72. Sodium Counter and Tracker for Healthy Food Diets	Apple	First Line Medical Communications Ltd	5/7/2017	4.99	NA	–	–	✔		✔					
73. Sodium Cravings – Control blood pressure with Sodium Tracker that manages salt food craving and enables healthy food choices	Apple	Blue Eyes Innovation LLC	17/6/2015	2.29	NA	–	–	✔	✔						

74. Sodium In Foods	Apple	Nassir Hussain	15/1/2018	Free	NA	–	–			✔					
75. Stress releaser	Apple	Jim Lu	20/11/2017	0.99	NA	–	–						✔		
76. Systolic - Blood Pressure Made Simple	Apple	Ford Parsons	-	Free	NA	–	–	✔			✔				
77. Tensio	Apple	humetrix.co m	31/10/2017	7.99	NA	–	–	✔		✔	✔			✔	✔
78. The Best Blood pressure monitor	Apple	A Cultivated Mindset, Inc	20/7/2015	2.29	NA	–	–	✔			✔				✔
79. Track my BP	Apple	New World Monkeys, LLC	21/8/2015	1.99	NA	–	–	✔							✔
80. ZOI Health - Your Personal Health Coach	Apple	PI Venture LLC, The	9/5/2017	9.99	NA	–	–			✔		✔			
81. ABC of Hypertension, 6th Edit	Google Play	Indextra AB	25/4/2017	16.99	1 - 5	–	–			✔					
82. Afya Pap	Google Play	Afya Pap	8/3/2017	Free	1,000 - 5,000	–	–	✔		✔					
83. Animated Atlas of Hypertension	Google Play	Focus Medica India Pvt. Ltd	23/11/2014	Free (10.99 - 21.23)[a]	5,000 - 10,000	–	–			✔					
84. Best Diet	Google Play	Moe Game	19/6/2017	Free	500 - 1,000	–	–			✔					
85. Best natural Herbs For High blood Pressure	Google Play	Obgynapps	19/11/2017	Free	1,000 - 5,000	–	–			✔					
86. Blood Pressure	Google Play	SiKni8	27/3/2016	0,59	10 - 50	–	–	✔		✔					
87. Blood Pressure (SmartBP)	Google Play	evolvemeds ys	8/11/2017	1.28	50,000 - 100,000	–	–	✔							✔
88. Blood Pressure Calculator	Google Play	Center for Research and Knowledge	2/6/2015	Free	500 - 1,000	–	–	✔	✔						
89. Blood Pressure Diary	Google Play	FRUCT	13/2/2018	Free (1.29 - 1.32)[a]	500,000 - 1,000,000	4.1	Y	✔	✔		✔				
90. Blood Pressure easy pro	Google Play	nockilllikout lindefol	-	Free	10,000 - 50,000	–	–	✔							
91. Blood pressure finger print scanner	Google Play	-	9/7/2017	Free	100 - 500	–	–	✔							
92. Blood Pressure Guide	Google Play	Anil	3/12/2014	Free	1,000 - 5,000	–	–			✔					

93. Blood Pressure Guide	Google Play	Nicholas Gabriel	20/11/2016	Free	500 - 1,000	–	–			✔					
94. blood pressure healthy Plus	Google Play	tdtomlou	7/2/2016	1.68	10 - 50	–	–	✔							✔
95. Blood Pressure Logger	Google Play	Time2Relax	18/10/2013	Free	5,000 - 10,000	–	–	✔			✔				✔
96. Blood Pressure Manager	Google Play	Nanjing Yuyue Software Technology Co.,Ltd	19/1/2018	Free	10 - 50	–	–	✔							
97. Blood Pressure manager	Google Play	ne.nemui	30/1/2016	Free	100,000 - 500,000	–	–	✔							✔
98. Blood Pressure Monitor	Google Play	Timevy	20/5/2017	Free	500 - 1,000	–	–	✔		✔					
99. Blood Pressure Monitoring	Google Play	Juan B and Juan H Android Developmen t	20/9/2015	Free	5,000 - 10,000	–	–	✔							✔
100. Blood Pressure Pro	Google Play	abletFactory	9/8/2013	2,38	100 - 500	–	–	✔							✔
101. Blood Pressure Symptoms	Google Play	vmappsoft.c om	-	Free	500 - 1,000	–	–			✔					
102. Blood Pressure Tracker	Google Play	aadhk	12/2/2017	8.99	100 - 500	–	–	✔							✔
103. Blood Pressure Tracker	Google Play	mallikarjunu du chittari	8/11/2017	Free	1,000 - 5,000	–	–	✔							✔
104. Blood Pressure(BP) Diary	Google Play	openit Inc.	6/9/2016	Free (1.49)[a]	1,000,000 - 5,000,000	3.8	Y	✔	✔		✔			✔	✔
105. Blood Pressure(BP) Report Lite	Google Play	TriStarApps	8/2/2012	Free	10,000 - 50,000	–	–	✔			✔				✔
106. BloodPressureDB	Google Play	Horst Klier	4/3/2017	Free	100,000 - 500,000	–	–	✔	✔						✔
107. BP Tracker	Google Play	Guangdong Biolight Meditech Co., Ltd.	19/9/2017	Free	1,000 - 5,000	–	–	✔		✔					
108. BP treatment no net	Google Play	abdo.apps	15/1/2018	Free	5,000 - 10,000	–	–			✔					
109. bpresso PRO	Google Play	Freshware	8/1/2017	4,89	1,000 - 5,000	4.4	N	✔	✔		✔			✔	✔
110. Braun Healthy Heart	Google Play	Kaz USA, Inc.	31/10/2015	Free	10,000 - 50,000	2.1	Y	✔	✔	✔	✔		✔		

111. Caracal	Google Play	Caracal	12/10/2017	Free	100 - 500	–	–	✔							✔
112. Cardio journal - Blood pressure diary	Google Play	mEL Studio	6/2/2017	Free (1.99 - 8.99)[a]	10,000 - 50,000	4.7	Y	✔	✔	✔	✔				✔
113. Complications of Hypertension	Google Play	Focus Medica India Pvt. Ltd	31/10/2017	Free (7.49)[a]	10 - 50	–	–			✔					
114. Control High Blood Pressure	Google Play	StatesApps	6/1/2018	Free	1,000 - 5,000	–	–			✔					
115. Control Tension	Google Play	Les Laboratoires Servier	6/4/2017	Free	-	NR	N	✔	✔	✔					✔
116. DASH Diet	Google Play	Chelin Apps	31/5/2017	Free	10,000 – 50,000	–	–			✔					
117. DASH Diet	Google Play	Nisarg D Parekh	1/1/2018	0.99	100 - 500	–	–			✔					
118. Dash Diet	Google Play	DashDietApps	18/3/2017	Free	100 - 500	–	–			✔					
119. Dash Diet	Google Play	DROPSOFT	4/10/2017	Free	50 - 100	–	–			✔					
120. DASH Diet 5 Days Meal Plan	Google Play	Gtype	12/2/2017	Free	500 - 1,000	–	–			✔					
121. Dash Diet Dynamite	Google Play	Nicole Woodinville	9/6/2016	Free	100 - 500	–	–			✔					
122. Dash Diet For Balanced Weightloss	Google Play	Diet Pundits	14/7/2016	Free	1,000 - 5,000	–	–			✔					
123. Dash Diet Guide	Google Play	Prestige Worldwide Apps, Inc	1/10/2015	Free	5,000 - 10,000	–	–			✔					
124. DASH Diet Meal Plan Recipes : Healthy, Weight Loss	Google Play	Edutainment Ventures-Making Games People Play	18/10/2017	Free (0.89 - 1.39)[a]	10 - 50	–	–			✔					
125. DASH Diet Plan	Google Play	Health Experts	26/3/2016	Free	10,000 - 50,000	–	–			✔					
126. DASH Diet Plan	Google Play	Bubble Shooting	14/7/2016	Free	500 - 1,000	–	–			✔					
127. Diabetes & Blood Pressure Log	Google Play	Cooey Technologies	30/3/2015	Free	100,000 - 500,000	–	–	✔	✔						✔
128. Diet Plan For Hypertension	Google Play	cvsilamedia.id	-	Free	1 - 5	–	–			✔					
129. Diet Plan Recipes	Google Play	hara5b68s	26/3/2015	Free	500 - 1,000	–	–			✔					
130. DynaPulse® PLUS	Google Play	Preventagen, Inc.	24/10/2016	Free	100 - 500	–	–	✔							

131. ESH CARE	Google Play	YouCo	15/11/2017	Free	10,000 - 50,000	2.5	N	✔	✔	✔					✔
132. Exercise Hypertension	Google Play	Built by Doctors World Ltd	19/12/2017	Free	100 - 500	–	–			✔					
133. Finger Blood pressure pro	Google Play	Nicolas Apps	5/1/2016	Free	10,000 - 50,000	–	–	✔							
134. Goal Achiever	Google Play	Techizer Tech Solutions Pvt Ltd	28/8/2017	Free	500 - 1,000	4.5	N	✔	✔	✔	✔				✔
135. Health Report Daily	Google Play	Elapse Technologie s	27/6/2017	1.89	10,000 - 50,000	–	–	✔							✔
136. Health, Nutrition & Diet Guide	Google Play	Organic Facts	16/1/2018	Free	100,000 - 500,000	–	–			✔					
137. Healthy Heart	Google Play	Neha Sethi	1/3/2017	Free	50 - 100	–	–			✔					
138. Heart Care - All In 1	Google Play	smart vue	23/9/2017	Free	100 - 500	–	–	✔		✔					
139. High Blood Pressure	Google Play	Free Apps For Everyone	31/5/2017	Free	100-500	–	–			✔					
140. High Blood Pressure	Google Play	bluebirdmed ia	5/1/2015	Free	500 - 1,000	–	–			✔					
141. High Blood Pressure	Google Play	Dev Galaxy Store	27/8/2017	Free	1 – 5	–	–			✔					
142. High Blood Pressure	Google Play	Personal Remedies LLC	15/6/2016	2,79	10 - 50	–	–			✔					
143. High Blood Pressure - The 101 Guide	Google Play	harrison.app s	282017/12/	Free	5-10	–	–			✔					
144. High Blood Pressure Diet Tips	Google Play	Data Recovery Software by RecoveryBu ll.com	20/2/2017	Free (2.19 - 21.99)[a]	10,000 - 50,000	–	–			✔					
145. High Blood Pressure In English	Google Play	COMPRINT	15/6/2016	Free	50 - 100	–	–			✔					
146. High Blood Pressure Pro	Google Play	showartzz	1/6/2016	Free	1 – 5	–	–			✔					
147. High Blood Pressure Symptoms	Google Play	Life Hack Studio	12/2/2018	Free	500 - 1,000	–	–			✔					
148. High Blood Pressure Symptoms	Google Play	Revolxa Inc	5/7/2017	Free	1,000 - 5,000	–	–			✔					
149. High Blood Pressure Symptoms	Google Play	Flames Dev Studio	5/4/2017	Free	10,000 - 50,000	–	–			✔					

150. High Blood Pressure Treatment	Google Play	shubh.techd	-	Free	1,000 - 5,000	–	–			✔					
151. High BP Hypertension Diet High Blood Pressure Help	Google Play	SendGroupSMS.com Bulk SMS Software	21/11/2017	Free 2.99 - 22.49)[a]	100 - 500	–	–			✔					
152. How To Lower Blood Pressure	Google Play	The Almighty Dollar	7/8/2017	Free	100 - 500	–	–			✔					
153. HyperHealth	Google Play	LES LABORATOIRES SERVIER	19/8/2016	Free	100 - 500	–	–			✔					
154. Hypertension	Google Play	MMI	9/9/2015	Free	100 - 500	–	–			✔					
155. Hypertension (An Overview)	Google Play	Focus Medica India Pvt. Ltd	26/10/2017	6.49	100 - 500	–	–			✔					
156. Hypertension Advisor	Google Play	shaon.cpp	16/5/2014	Free	1,000 - 5,000	–	–	✔			✔				
157. Hypertension and treatment	Google Play	mircica	-	0.85	10 - 50	–	–	✔			✔				
158. Hypertension Drugs Dictionary	Google Play	alexeykharchenko	-	Free	50,000 - 100,000	–	–			✔					
159. Hypertension guideline check	Google Play	academy.de	-	Free	1,000 - 5,000	–	–	✔			✔	✔			
160. Hypertension Hi blood pressure	Google Play	twayesh	-	Free	100,000 - 500,000	–	–			✔					
161. Hypertension Management	Google Play	wonderfullife.develloper	-	Free	1,000 - 5,000	–	–	✔							
162. Hypertension management	Google Play	Benson Media	24/2/2017	Free	1,000 - 5,000	–	–			✔					
163. Hypertension Protocols	Google Play	Dr.Isaac's Holistic Wellness	3/12/2017	Free	500 - 1,000	–	–			✔					
164. Hypertension Symptoms	Google Play	ketopoxstudio.inc	18/6/2016	Free	5-10	–	–			✔					
165. Hypertension Treatment JNC 8	Google Play	ahscbc	-	Free	10,000 - 50,000	–	–	✔			✔				
166. iBP Blood Pressure	Google Play	Leading Edge Apps LLC	30/11/2014	0,62	10,000 - 50,000	–	–	✔			✔				✔
167. KKM/BKP Hypertension CPG QR	Google Play	KKM/BKP	30/12/2014	Free	100 - 500	–	–			✔					
168. Know Hypertension	Google Play	Dr. Rabi Ekore	22/2/2017	Free	100 - 500	–	–			✔					

169. LifeCourse HyTen	Google Play	PInCH Medical Systems Limited	26/10/2016	Free	1 – 5	5.0	Y	✔	✔	✔	✔				
170. Lower Blood Pressure Foods	Google Play	FreeAppsForAll	25/3/2016	Free	5,000 - 10,000	–	–			✔					
171. Manage your Hypertension five	Google Play	epat.mobi	3/1/2017	Free	50 - 100	–	–	✔		✔					
172. Manage Your Hypertension Four	Google Play	epat.mobi	-	Free	50 - 100	–	–		✔	✔					
173. Manage your Hypertension six	Google Play	Epat Lda	7/12/2016	Free	50 - 100	–	–			✔					
174. Manuel hypertension artérielle	Google Play	TEMPO MEDICAL INTERNATIONAL	7/12/2015	Free	1-5	–	–			✔					
175. My Food Coach	Google Play	National Kidney Foundation	1/3/2016	Free	10,000 - 50,000	–	–			✔					
176. My heart	Google Play	Klimaszewski Szymon	25/1/2018	Free (1.29 - 3.09)[a]	5,000,000 - 10,000,000	4.2	Y	✔	✔		✔				✔
177. Nutrition Hypertension	Google Play	Built by Doctors World Ltd	9/11/2016	Free	100 - 500	–	–			✔					
178. Overcome hypertension	Google Play	fejriidroid	28/3/2017	Free	100 - 500	–	–			✔					
179. Paracelsus (Pressure control)	Google Play	Medical software of Ukraine	3/10/2016	Free	5,000 - 10,000	3.7	Y	✔	✔	✔					✔
180. Pukono	Google Play	Fundació Althaia	12/7/2017	Free	10,000 - 50,000	–	–			✔					
181. Qardio Heart Health	Google Play	Qardio, Inc.	14/12/2017	Free	100,000 - 500,000	4.4	Y	✔	✔		✔				
182. Reducing Blood Pressure	Google Play	Quilckredapps	27/2/2016	Free	50 - 100	–	–			✔					
183. SA Hypertension	Google Play	Appenberg Digital Publishing	15/1/2016	Free	100 - 500	–	–			✔					
184. Tips for Lowering High Blood	Google Play	sidikdroid	NR	Free	1,000 - 5,000	–	–			✔					
185. Tips to Overcom High Naturally	Google Play	Sutriyanidroid	23/12/2016	Free	500 - 1,000	–	–			✔					
186. Vital Tones Hypertension Pro	Google Play	Vital Tones	13/12/2017	9.99	5 - 10	–	–			✔			✔		

[a] The app was free to download, but require subscription fees; [b]NR, not reported; [c]NA: not applicable; [d]_: the privacy assessment was not carried out/ the app user rating was not extracted; [e]Y: the app had an available privacy policy without the need to download it; [f]N: the app did not have a privacy policy.
